# The curvHDR method for gating flow cytometry samples

**DOI:** 10.1186/1471-2105-11-44

**Published:** 2010-01-22

**Authors:** Ulrike Naumann, George Luta, Matthew P Wand

**Affiliations:** 1 School of Mathematics and Statistics, The University of New South Wales, Sydney 2052, Australia; 2 Department of Biostatistics, Bioinformatics, and Biomathematics, Georgetown University Medical Center, Washington, DC 20057-1484, USA; 3 School of Mathematics and Applied Statistics, University of Wollongong, Wollongong 2522, Australia

## Abstract

**Background:**

High-throughput flow cytometry experiments produce hundreds of large multivariate samples of cellular characteristics. These samples require specialized processing to obtain clinically meaningful measurements. A major component of this processing is a form of cell subsetting known as *gating*. Manual gating is time-consuming and subjective. Good automatic and semi-automatic gating algorithms are very beneficial to high-throughput flow cytometry.

**Results:**

We develop a statistical procedure, named curvHDR, for automatic and semi-automatic gating. The method combines the notions of significant high negative curvature regions and highest density regions and has the ability to adapt well to human-perceived gates. The underlying principles apply to dimension of arbitrary size, although we focus on dimensions up to three. Accompanying software, compatible with contemporary flow cytometry infor-matics, is developed.

**Conclusion:**

The method is seen to adapt well to nuances in the data and, to a reasonable extent, match human perception of useful gates. It offers big savings in human labour when processing high-throughput flow cytometry data whilst retaining a good degree of efficacy.

## Background

Flow cytometry is a laser-based biotechnology that produces large multivariate samples. Typically, each member of the sample corresponds to the physical properties of a biological cell - known as *forward scatter *and *side scatter *- and antibody binding activity, through fluores-cence intensity measurements. The latter measurements arise from the cells being exposed to several fluorescently conjugated antibodies during the flow cytometry procedure. Shapiro [1] provides a detailed summary of flow cytometry technology and its practice.

The last few years have seen a major change in flow cytometry technology, toward what has become known as *high-throughput *flow cytometry or *high-content *flow cytometric screening (FC-HCS) (e.g. Le Meur *et al*. [2]). FC-HSC combines robotic fluid handling, flow cytometric instrumentation and bioinformatics software so that relatively large numbers of flow cytometric samples can be processed and analysed in a short period of time. Currently, analysis of such data involves a tremendous amount of manual manipulation. This is costly in time and human energy, and renders the analysis more subjective and error-prone. An early article on FC-HCS by Gasparetto *et al*. [3] closes with: "Further improvements that completely automate the FC-HCS procedures and incorporate newly developed advanced data analysis and management features will further improve the efficiency and power of this technique".

An integral component of flow cytometric data analysis is *gating*, where cells are subsetted according to physical and fluorescence measurements. Recent studies involving high throughput flow cytometric data (e.g. Gasparetto *et al*. [3]; Brinkman *et al*. [4]) have involved manual gating of hundreds of flow cytometric samples. Automatic gating methods are becoming more important in contemporary flow cytometry research. If done well, they are more objective, much faster and less expensive. Combined with the automated aspects of new high-throughput flow cytometry technology good automatic gating methods have the potential to open up a wide range of possibilities in biomedical research.

In this article we describe a new method for automatic and semi-automatic gating of multivariate flow cytometry samples. We call the method curvHDR since it makes use of two statistical concepts with regard to the density of the samples: (a) significant high negative curvature corresponding to modal regions and (b) highest density regions (HDR) for data in the vicinity of identified modal regions. The significant curvature phase is useful for identifying regions containing a possibly interesting subset of cells. The HDR phase then aims to improve upon high curvature regions and mimic human perception of what are subsets of interest. The principles underlying curvHDR apply to samples of arbitrary dimension. However, in the present article, we restrict attention to dimensions between one and three.

Often the gate obtained from curvHDR needs to be combined with other simpler gates for effective utilisation. One instance where this applies is when unimportant 'debris' cells near the boundary of the sample exhibit high negative curvature in their density. Rectangular gating, where variables in each direction are restricted to lie within an interval, is often an effective means of eliminating spurious components of a curvHDR gate. Naumann & Wand [5] used curvHDR gates combined with rectangular gates in a flow-cytometric application. The Results section provides some illustration of this type of gating.

Our curvHDR methodology is accompanied by software in the R computing environment (R Development Core Team [6]) and, hence, can be integrated into Bioconductor (Gentleman *et al*. [7]).

The ability to handle trivariate samples is a particularly novel aspect of curvHDR. Traditionally, gating has been limited to two dimensions because of graphical display restrictions. However, recent developments in three dimensional (3D) graphics in the R computing environment allow for routine visualisation of trivariate data and polyhedral gates. The R packages rgl (Adler & Murdoch [8]) and misc3d (Feng & Tierney [9,10]) are especially useful for work of this kind.

Not surprisingly, other research teams involved in flow cytometric data analysis recently have been developing automatic gating procedures in response to the high-throughput sea change. For example, Lo, Brinkman & Gottardo [11] combine t-mixture models and Box-Cox transformations to obtain flexible and outlier-resistant gates whilst Finak, Bashashati, Brinkman & Gottardo [12] use the Bayesian Information Criterion to approximate optimal merging of such gates. In our view, it is too early for extensive comparison of automatic gating procedures that have been spawned by the demands of high-throughput flow cytometry. At this stage we welcome the development of a variety of approaches. Detailed comparative evaluation would be useful at a later stage; after the 'dust settles'. However, the Results section contains some very brief comparison of curvHDR with the method of Lo *et al*. [11].

### Flow cytometry background

Shapiro [1] provides a comprehensive survey of flow cytometry. Mathematically, typical flow cytometric samples can be thought of as large point clouds in high-dimensional space. The dimension is somewhere between about 3 and 15 and the number of points, usually corresponding to cells, is often between tens of thousands and hundreds of thousands. Two of the dimensions usually correspond to the intensity of *forward scatter *and *side scatter *which characterise the physical properties of the cell (e.g. size and granularity). The remaining dimensions correspond to the intensity of the cell's fluorescence at a given wavelength (colour). In medical research contexts the colours often correspond to staining of the cells by monoclonal antibodies.

The most important types of gating are (i) bivariate cell-type gating (e.g. identification of lymphocytes from scatterplots of forward-scatter versus side-scatter measurements) and (ii) univariate fluorescence-channel gating (e.g. identification of cells that recognise a particular antibody). However, there is no cogent reason for restriction of gating to one- and two- dimensional projections of flow cytometry point clouds. Roederer & Hardy [13], for example, advocate gating in three and higher dimensions.

Manual gating in practical flow cytometry data analyses usually involves a combination of biological domain knowledge and visual inspection of flow cytometry scatterplots and histograms. But, typically, gates correspond to *modal regions *in the data. Mathematically, modal regions are those regions where the underlying density function of the data is higher than surrounding regions. The quality of an automatic gating method depends on how well it mimics human perception of what is an appropriate gate. Obviously, this is a difficult goal since perceptions differ from one human to another and there is no single 'right answer'.

**Figure 1:**
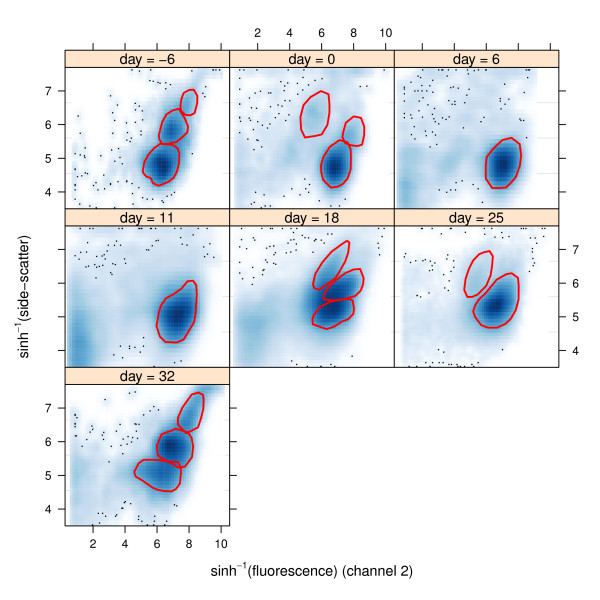
**Illustration of bivariate manual gating by a flow cytometry expert: Dr John Zaunders of the Centre for Immunology, Sydney, Australia**. The gates correspond to the red-coloured shapes. The flow cytometry data correspond to a study on graft-versus-host disease (source: Brinkman *et al*. [4]). The panels correspond to day number with respect to blood and marrow transplant of a particular patient. The vertical axis is sinh^-1^(side-scatter), whilst the horizontal axis is sinh^-1^(fluorescence) for the second channel. Since the data are large flowViz defaults to displaying the data as smoothed scatterplots, based on bivariate kernel density estimation.

An appreciation of human-perceived gates can be obtained from Figure 1. The data are an illustrative subset of the longitudinal flow cytometric data on graft-versus-host disease described in Brinkman *et al*. [4] and are available in the Bioconductor package flowViz (Ellis *et al*. [14]; Sarkar, Le Meur & Gentleman [15]) where it is stored as a *flowSet *named GvHD. Each panel corresponds to a different day number with respect to blood and marrow transplant of a particular patient. The vertical axis is sinh^-1^(side-scatter) whilst the horizontal axis is sinh^-1^(fluorescence) for the second channel. The gates were drawn by a flow cytometry expert: Dr John Zaunders of the Centre for Immunology, Sydney, Australia. At the time that the gates were drawn, Dr Zaunders had no knowledge of the present article or its content.

Manual gates such as those shown in Figure 1 combine biological domain knowledge with the modal regions apparent from the data. The former is not easily quantified mathematically. Nevertheless, automatic and semi-automatic gating that makes use of the modal region aspects of gating can still be very useful: taking away the human judgement element and permitting faster processing of high-throughput samples. The curvHDR method, described in the next section, aims to fill this niche.

## Methods

Let *d *be the dimension of data in which a gate is sought and let

be a sample in ℛ ^*d *^for which gating is desirable. We will assume that gates of interest correspond to *modal regions *in the sample. This first entails assuming that the ***x***_*i*_s are a sample from a smooth *d*-variate density function *f*. Modal regions then correspond to local maxima in *f *and their surrounds.

The first phase of the curvHDR method employs recently developed feature significance technology (Duong, Cowling, Koch & Wand [16]) to find regions where *f *has statistically significant high negative curvature. This phase can be thought of as filtering process where aberrant regions of high relative density are ignored and only those regions having statistical evidence of modality are retained. The second phase aims to improve upon the regions obtained in the first phase by modifying them to suit the local density of the data around each high curvature region.

The specific steps of the curvHDR gating method are:

(1) Remove excessive boundary points and other debris from the data. If the data exhibits heavy skewness then transform the data to reduce skewness. A good 'all-purpose' transformation is the inverse hyperbolic sine transformation .

(2) Standardise all variables to have zero mean and unit standard deviation.

(3) Obtain significant high negative curvature regions using the test described in Section 3.2 of Duong *et al*. [16] over a *d*-dimensional mesh. The regions are stored as intervals for univariate data (*d *= 1), polygons for bivariate data (*d *= 2) and polyhedra for trivariate data (*d *= 3). Let *S *denote the number of significant curvature regions.

(4) Replace each of the *S *significant curvature regions by their convex hulls.

(5) Grow each convex hull so that its volume is *G *times larger (for some pre-specified growth factor *G *> 1). This is achieved by 'rolling' a *d*-dimensional sphere around the perimeter of the region.

(6) For each of the *S *grown regions, determine the subset of the data lying inside that region.

(7) For each of the *S *data subsets, obtain a kernel density estimate, based on a multistage plug-in bandwidth selector (Duong & Hazelton [17]), and using only the data in that subset.

(8) The curvHDR gate is the union of the level-*τ *HDRs (see definition below) based on the *S *kernel density estimates. The curvHDR gate will have greater than or equal to *S *components, where a component is an interval, polygon or polyhedron depending on whether *d *= 1, *d *= 2 or *d *= 3.

(9) Determine the indices of the data corresponding to the curvHDR gate.

(10) Transform the gate and gated data back to the original units.

**Figure 2:**
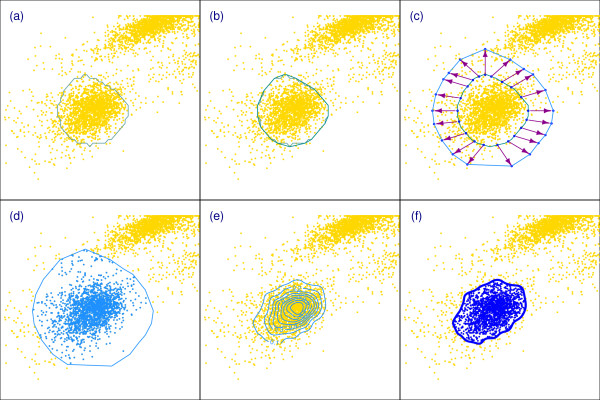
**Graphical illustration of curvHDR gating for bivariate data**. **Panel (a): Polygon corresponding to a region of statistically significant high negative curvature**. Panel (b): The convex hull of the polygon from (a). Panel (c): A new, larger, polygonal region obtained by growing the region from (b) using the notion of 'sphere rolling' (in this bivariate case it is 'circle rolling') around inner polygon. Approximate circle rolling is achieved by taking normal vectors of equal length from the centre of each edge of the inner polygon. The size of the outer polygon is chosen so that the ratio of its area to the inner polygon is a pre-specified growth factor *G*. Panel (d): The bivariate measurements are subsetted according to inclusion inside the polygon from (c). Panel (e): A kernel density estimate is obtained using only the subsetted data from (d). Panel (f): The final gate corresponds to a high density region contour of the kernel density estimate from (e), in this case the *τ *= 0.1 highest density region.

Figure 2 provides graphical illustration of Steps (3)-(8) for the case *d *= 2.

Step (3) requires estimates of the Hessian matrix of *f*, the *d *× *d *matrix with (*i, j*) entry equal to , with *x*_*i *_denoting the *i*th entry of ***x***. Each derivative estimate is obtained via appropriate differentiation of the *d*-variate kernel density estimator(1)

where *K *is a *d*-variate kernel function and ***H ***is a *d *× *d bandwidth matrix*. Details are given in Duong *et al*. [16]. In curvHDR we use a single parameter bandwidth matrix  for some *h*_curv _> 0. This is partially justified by the fact that input data for kernel density estimation is such that each variable has unit standard deviation. Several embellishments are possible, each covered by Wand & Jones [18], but are yet to be entertained for curvHDR. Section 3.2 of Duong *et al*. [16] describes how the estimated Hessian matrix can be used to determine regions in ℛ^*d *^where *f *has significant high negative curvature. These correspond to local maxima in the underlying density and identify candidate locations for which gating might be appropriate.

The R package feature (Duong & Wand [19]) provides implementation of the significant curvature determination. Efficient computation is achieved using linear binning over a *d*-variate grid (Wand [20]). This approach leads to a grid of indicators (0/1) for significant high negative curvature. Contouring functions in R such as contourLines() in bivariate case and contour3d() in the trivariate case can then be used to extract and store the regions as polygons (*d *= 2) or polyhedra (*d *= 3). The *d *= 1 case is much simpler and high curvature regions correspond to intervals.

Details on Steps (4)-(6) are postponed to upcoming subsections, where the *d *= 2 and *d *= 3 cases are treated separately. No such details are necessary for *d *= 1 since these steps involve elementary manipulations of intervals.

Step (7) involves application of formula (1) to each grown region and the data that it contains. The kernel *K *is taken to be the *d*-variate standard normal density function

The bandwidth matrix is chosen using multi-stage plug-in strategies (Duong & Hazelton [17]; Wand & Jones [21]) courtesy of the R package ks (Duong [22]). Further details are given in the parameter choice subsection. In most cases, the Step (6) density estimates are concerned with unimodal structure where plug-in bandwidths perform quite well.

For a *d*-variate density function *f *and *τ *∈ [0, 1] the *τ *highest density region (HDR) is

(e.g. Hyndman [23]). We can think of the *R_τ _*as corresponding 'meaningful' contours of the density function *f*. For example, *R*_0.9 _is the region inside that contour of *f *for which the probability is 0.1, a relatively small region near the peak of *f*. The HDR *R*_0.1 _encompasses 90% of the probability mass of *f*. In practice, where *f *is unknown, estimated HDRs can be obtained by replacing *f *with a density estimate.

In Step (8) we apply the HDR paradigm to each of the density estimates from Step (7). Typically, *τ *is fixed for all regions although individual *τ *values could also be specified. We have found that lower *τ *values are more in keeping with human-based gating.

Step (9) is similar to Step (6), and details of its execution are discussed in subsections devoted the additional details for bivariate and trivariate samples.

### Additional details for bivariate samples

In this section we provide details on aspects of the curvHDR method that are specific to the bivariate case. We begin with Figure 2, which provides a visual overview of curvHDR when *d *= 2.

We now give some details on Steps (4)-(6) in the *d *= 2 case, as displayed in Panels (b)-(d) of Figure 2.

The convex hull of a polygon in ℛ^2 ^is a well-known geometrical construct. A useful physical interpretation involves imagining the vertices of the polygon as nails on a board and stretching an elastic band around outside of the nails. The convex hull then corresponds to the stretched elastic band. In R the convex hull of a polygon can be obtained using the base function chull().

Step (5) involves growing a convex polygon to be *G *times larger in area via the notion of 'circle-rolling'. We first note that the area of a polygon with vertices

and ordered clockwise and such that (*x*_1_, *y*_1_) = (*x*_*N*_, *y*_*N*_) is

Now suppose that we roll a circle of radius *r *around the perimeter of . A polygonal approximation to the resulting region is obtained by forming normal vectors to each edge of  that start from the centre of the edge and radiate outwards a distance of 2*r*. This approach is illustrated in Panel (c) of Figure 2. Let  denote the polygon obtained by joining each of the normal vectors. Step (5) is completed by solving for the *r *that satisfies *A*()/*A*() = *G*. In our implementation of curvHDR we use a simple bisection search to determine *r*.

Steps (6) and (9) require the determination of those points that are inside a particular polygon. This is a relatively simple geometric problem and implemented in R by a number of packages. Flow cytometric sample sizes are quite large and speed is important. For this reason, we recommend the function inpolygon() from the Bioconductor package flowCore (Ellis, *et al*. [24]).

All bivariate kernel density and curvature estimates are obtained via the binned approximation (Wand [20]) over a fine mesh. Choice of the bandwidth matrix is discussed in the parameter choice subsection.

### Additional details for trivariate samples

In three dimensions the convex hull corresponds to 'shrink wrapping' a closed polyhedron, and is required for Step (4). Trivariate convex hull computation is facilitated by the function convhulln() in the R package geometry (Grasman & Gramacy [25]).

Steps (3) and (4) make use of the three-dimensional contour functionality in the R package misc3d (Feng & Tierney [9,10]). This package uses *triangle mesh objects *for storing and displaying polyhedra. The faces of such polyhedra are triangles. For triangular-faced poly-hedra, Step (5) is relatively straightforward. A polyhedron is grown by placing a sphere of radius *r *tangentially to each triangular face, and touching the face at the triangle's centroid. The new polyhedron is the convex hull of the set of antipoles of the touching points. The value of *r *is chosen so that *V*()/*V*() = *G*, where *V *() is the volume of an original polyhedron (obtained in Step (4)) and *V *() is the volume of the grown polyhedron. Note that convhulln() has an option to compute the required volumes.

Steps (6) and (9) require determination of those points in a trivariate sample that lie inside a given polyhedron. This is a non-trivial problem and, to the best of our knowledge, is not supported by any of the current R packages on the Comprehensive R Archive Network. We use an efficient algorithm, specifically designed for large-scale problems that involve testing if a large number of points (e.g. hundreds of thousands) lie inside a triangular-faced polyhedron, composed itself of many vertices and faces. The basic idea of the algorithm is that only some faces of the triangular mesh are needed to perform the point containment test; after one of these determining faces is found, testing the given point against this face is sufficient to determine if the point lies inside or outside of the general polyhedron. It should be noted that in principle the algorithm can be extended to higher dimensions. A C++ implementation of this recently developed algorithm is available at the web-site. http://ptinpoly.pbwiki.com.

### Parameter choice

The curvHDR gating method has a suite of parameters that need to be either set to reasonable defaults or chosen by the user. In the interests of making curvHDR as automatic as possible we have, based on extensive experimentation, determined defaults for most of those parameters with the intention that they can remain in the 'background'. Table 1 summarises these default choices.

**Table 1:** Recommended defaults for curvHDR

parameter	default
bandwidth for significant curvature phase (*h*_curv_)	[4/{(*d *+ 6)*n*}]^1/(*d*+8)^
significance level for significant curvature phase	0.05
growth factor (*G*)	2^*d*^
bandwidth matrices for the HDR phase	multi-stage plug-in

The bandwidth for the significant curvature phase is the optimal bandwidth for estimation of the *d*-variate Hessian matrix when *f *is the standard normal density (Chacon, Duong & Wand [26]). Since the Gaussian density is close to being that with the largest optimal amount of smoothing (Terrell [27]), the table entry corresponds, approximately, to the biggest bandwidth that should be considered for curvature estimation. Note that this formula is only appropriate when the data have first been standardised to have unit standard deviation - as dictated by Step (2).

The curvHDR gate is relatively insensitive to the choice of the significance level for the significant curvature phase and any small value of this parameter is likely to be adequate. Our recommendation of 0.05 matches the most common default for a significance level in statistical procedures.

The growth factor *G *is defaulted to 2^*d *^since it corresponds to an approximate doubling of the size of the original region in each dimension, and has given reasonable answers in examples that we have studied to date. However, there may be circumstances where smaller or larger *G *values are required for curvHDR to match human-perceived gates.

Recall that Step (7) involves computation of *S d*-variate kernel density estimates: one for each subset obtained in Step (6). Ideally, these density estimators would use bandwidth matrices tailored for HDR estimation. At the time of this writing, there are no such bandwidth selection algorithms for general *d*; although Samworth & Wand [28] have recently treated the *d *= 1 version of the problem. Given its good simulation performance, and because of its availability in R, our current recommendation is to use the multi-stage plug-in bandwidth selector of Duong & Hazelton [17]. This is available in the R package ks (Duong [22]). For *d *= 1 the relevant function is hpi() while for *d *= 2, 3 it is Hpi.diag(). For flow cytometric data it is important that the binning flag is set to TRUE since, without binning, the computation is unacceptably slow. Note that ks currently only supports binning for diagonal bandwidth matrices. Finally, for speed reasons again, in the *d *= 3 case it is recommended that Hpi.diag() uses pilot="samse" and the binning mesh size be kept at a low value such as 21 × 21 × 21.

The only parameter not listed in Table 1 is the level of the highest density region *τ *. This is because we are uncomfortable about setting a default, given that perception of what is a reasonable gate is somewhat fuzzy, and differs between analysts. Therefore, *τ *is the main tuning parameter of curvHDR and it is recommended that the user experiments with its choice, perhaps in combination with changes in *G*. However, if pressed for a default, then *τ *= 0.1 is a somewhat reasonable answer.

### Software

We have written an R function named curvHDRfilter() for implementation of the curvHDR algorithm for input data having dimension between one and three. An accompanying plot() function allows visualization of the gates. For trivariate data, visualization is aided by the RGL graphics device and the packages rgl (Adler & Murdoch [8]) and misc3d (Feng & Tierney [9,10]). We have commenced work with the developers of flowCore (Ellis *et al*. [24]) towards making curvHDR usable within that environment. Meanwhile, packaged code and an accompanying vignette is available from the third author (current e-mail address: mwand@uow.edu.au).

## Results

We will now provide illustration of curvHDR on the longitudinal flow cytometric data of Figure 1. With space constraints and pedagogy in mind, the illustrations are kept simple and distinct from clinical interpretation and outcomes. See Naumann & Wand [5] for application of curvHDR to cellular signature determination for the graft-versus-host disease data.

### Illustrations of univariate curvHDR

**Figure 3:**
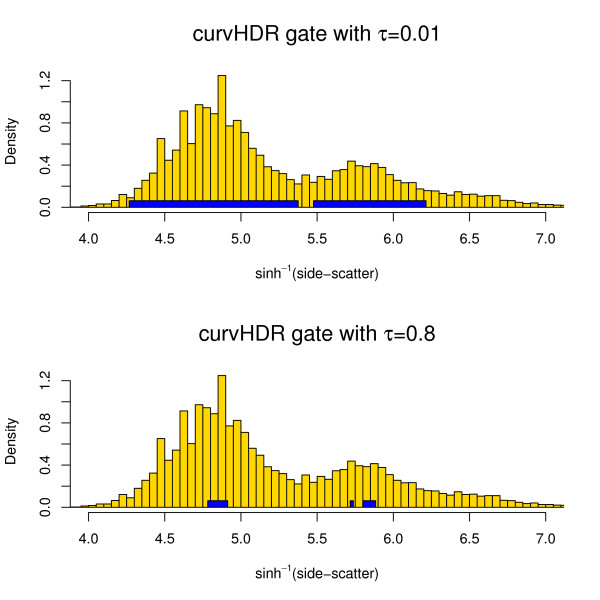
**Examples of univariate curvHDR gates with HDR levels set at *τ *= 0.01 and *τ *= 0.8**.

Figure 3 shows two univariate curvHDR gates for some side-scatter data from the GvHD flowSet. The data and its histogram can be obtained using the R commands:

library(flowViz) ; data(GvHD)

inputData <- asinh(exprs(GvHD$s9a01)[,2])

hist(inputData,breaks = 100,xlim = c(4,7))

The curvHDR gate in the upper panel has the HDR level set at *τ *= 0.01, whilst the lower panel has *τ *= 0.8. The *τ *= 0.01 gate consists of two intervals; the *τ *= 0.8 consists of three intervals.

### Illustrations of bivariate curvHDR

**Figure 4:**
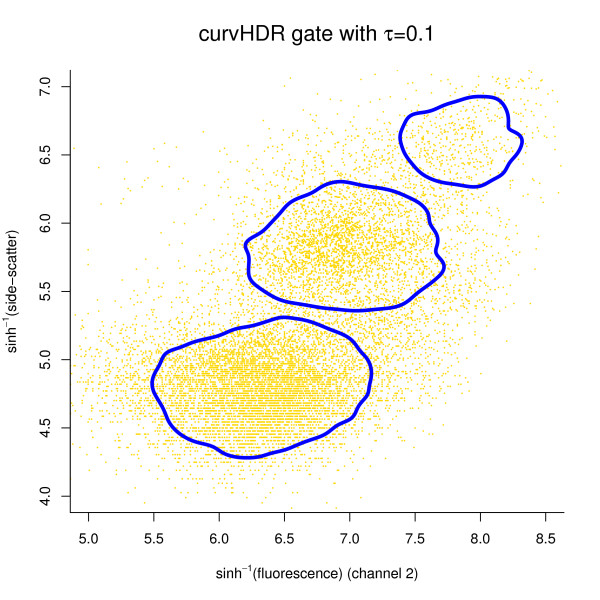
**curvHDR gate of data in the upper-left panel of Figure 1 (corresponding to 6 days before transplant)**. The HDR level parameter is equal to 0.1.

Figure 4 shows a bivariate curvHDR gate with *τ *= 0.1. The data are those shown in the upper left-hand panel of Figure 1, corresponding to 6 days before transplant. The data and corresponding scatterplot can be obtained using the R commands:

library(flowViz); data(GvHD)

inputData <- asinh(exprs(GvHD$s9a01) [,c(4,2)])

plot(inputData[,1,inputData[,2,xlim = c(5,8.5),ylim = c(4,7))

In Figure 4 we have plotted a subset of these data to enhance visualisation.

**Figure 5:**
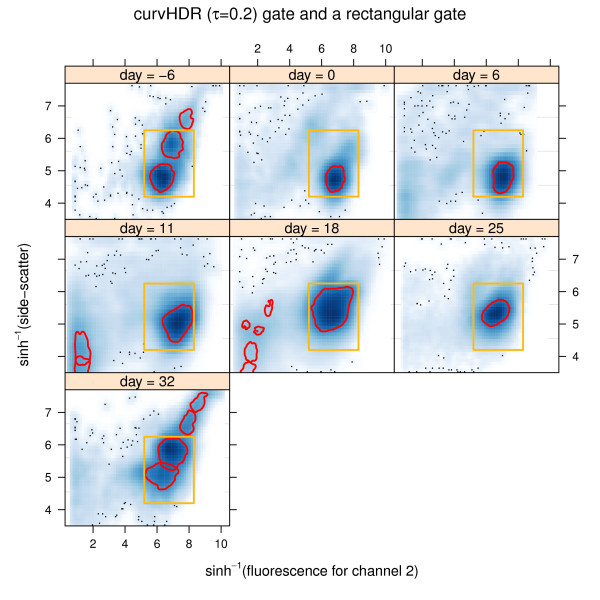
**The result from applying curvHDR gates (with *τ *= 0.2) to data corresponding to each panel of Figure 1**. The rectangle in each panel is that given by (2).

Figure 5 shows the result of applying *τ *= 0.2 gates to all 7 scatterplots. In practice, it is often desirable to restrict attention to a sub-region of the data. An effective means of doing this is via intersection with a rectangle. The rectangles in Figure 5 correspond to(2)

Figrure 6 shows the gates after intersection with the rectangle. We call these *rectangle-curvHDR *gates.

**Figure 6:**
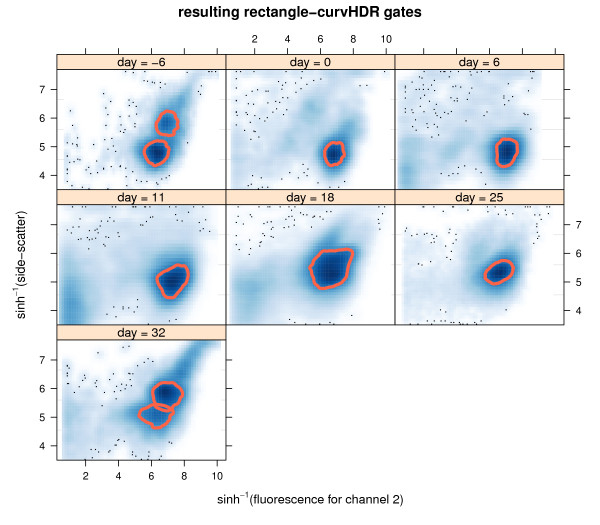
**The resulting rectangle-curvHDR gates, obtained from Figure 5 by intersecting each of the rectangle-curvHDR gates with the rectangle (2)**.

Comparison of Figure 6 with Figure 1 reveals some resemblance between the curvHDR gates and the manual gates that curvHDR is striving to emulate. However, there are also some noticeable differences, as seen by comparing the day = 18 panels. This illustrates limitations of mode-based automatic gating methods. A fuller comparison would be interesting, but should involve gates from several experts choosing gates for a larger number of data-sets, as well as different choices of the curvHDR tuning parameters.

### Comparison with flowClust

**Figure 7:**
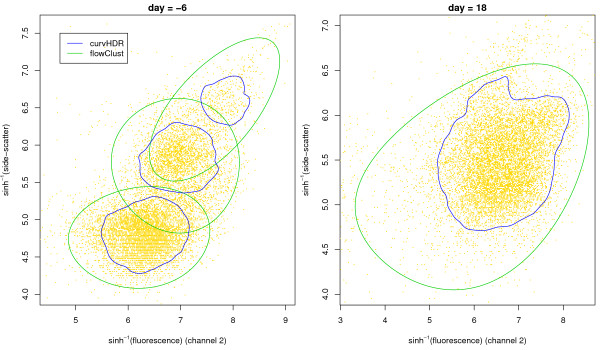
**Visual comparison of curvHDR with flowClust**. The data correspond to day = -6 and day = 18 from Figure 5.

The Bioconductor package flowClust (Gottardo and Lo [29]; Lo, Hahne, Brinkman and Got-tardo [30]) achieves automated gating through the use of t-mixture models and Box-Cox transformations. Details of the methodology may be found in Lo, Brinkman & Gottardo [11]. Figure 7 facilitates a cursory visual comparison of curvHDR with flowClust. The data correspond to day = -6 and day = 18 from Figure 5. All gates correspond to the default settings of the tuning parameters. For curvHDR the defaults correspond to Table 1 and *τ *= 0.1. For flowClust the defaults in its Bioconductor implementation were used. The flowClust method requires specification of the number of clusters *K*. We set *K *= 3 for the day = -6 data and *K *= 1 for the day = 18 to match the number of polygons found by curvHDR, excluding sparse data boundary regions.

One important difference, apparent from Figure 7, is that curvHDR is *nonparametric*, without any particular shape restrictions, whilst the flowClust gates are *parametric *- i.e. they correspond to inverse Box-Cox transformations of ellipses. The nonparametric aspect of curvHDR allows it to better adapt to the modal structure in the flow cytometry data. The day = 18 data exhibits a pronounced non-convex modal region, and this is captured by the curvHDR gate. For the day = -6 the curvHDR is more focussed, with non-overlapping gates for each of the modal regions. The flowClust gates are centred on the same regions, but are considerably larger and overlapping.

### Illustrations of trivariate curvHDR

We now provide an illustration of trivariate curvHDR by adding a third variable, forward-scatter, to the longitudinal data of Figure 5. The data and corresponding scatterplot can be obtained using the R commands:

library(flowViz) ; data(GvHD)

inputData <- asinh(exprs(GvHD$s9a01) [,c(1,2,4)])

We combined *τ *= 0.5 curvHDR gating with the rectangular gate:(3)

**Figure 8:**
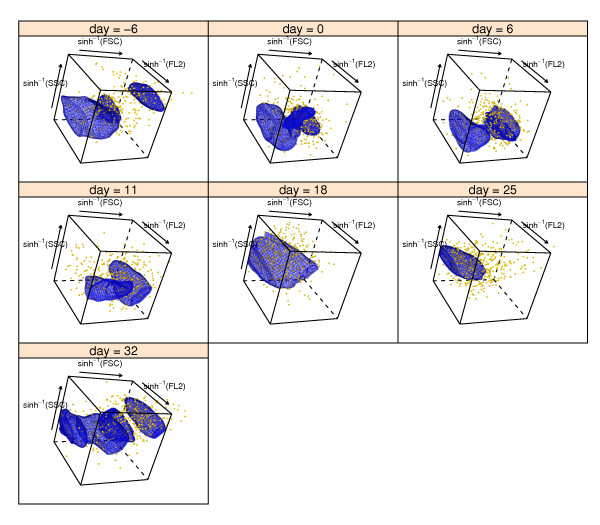
**Illustration of trivariate rectangle-curvHDR gating**. The data are the same as in Figures 5 and 6, but with sinh^-1^(forward-scatter) added as a third variable. The rectangular gate is given by (3). The axis labels use the abbreviations: FSC for forward-scatter, SSC for side-scatter and FL2 for fluorescence from channel 2.

The resulting rectangle-curvHDR gates are shown in Figure 8. Note that each of the gates consist of between 1 and 3 polyhedra.

Semi-automatic trivariate gating is a novel concept for flow cytometric data analysis. Just as the bivariate gating can offer improvements over univariate gating, we anticipate benefits arising from trivariate gating. With the advent of good three-dimensional visualisation software in R/Bioconductor and the emergence of trivariate gating algorithms, such as curvHDR with *d *= 3, we envisage flow cytometry data analysis breaking away from its current custom of restricting views and gates to two dimensions.

## Discussion

The curvHDR method is an intuitive and reasonably simple mechanism for obtaining candidates for cell-type gating. The method is intrinsically non-parametric, allowing it to adapt to the data without the restrictions of parametric methods such as those based on the Gaussian density function. Consequently the curvHDR regions are not restricted to be ellipsoidal or to have some other regular shape. With judicious choice of the main tuning parameter *τ*, possibly in combination with the secondary tuning parameter *G*, it can mimic human gating quite well. In combination with simple rectangular gating it provides a powerful base with which to build effective automatic gating strategies.

Whilst we have restricted attention and software development to dimensions 1-3 there is no firm upper limit on the dimensionality in which curvHDR can be applied. Extension of curvHDR beyond three dimensions, in terms of practicable algorithms and software, is an interesting new research problem - and one which could be quite fruitful as flow cytometric data becomes more abundant and complex.

## Conclusions

In this study we have proposed an automatic gating method named curvHDR, and worked out the algorithmic details for univariate, bivariate and trivariate data. The method is seen to adapt well to nuances in the data and, to a reasonable extent, match human perception of useful gates. Naumann & Wand [5] have already used curvHDR in a high-throughput flow cytometry application and demonstrated its efficacy, with big savings in human labour. We are in the process of incorporating the methodology into the R/Bioconductor computing environment with flowCore compatability. This will facilitate the use of curvHDR in future high-throughput flow cytometry analyses.

## Authors' contributions

UN contributed to development of the methodology and programmed the univariate and bivariate versions of the algorithm. GL contributed to development of the methodology and programmed the trivariate version of the algorithm. MPW contributed to development of the methodology, coordinated the study and finalized the manuscript. All authors read and approved the final manuscript.
